# Supplementary material for: Ultra-high thermal stability of sputtering reconstructed Cu-based catalysts
Source: Nat Commun. 2021 Dec 10;12:7209. doi: 10.1038/s41467-021-27557-1 (PMC8664808; doi:10.1038/s41467-021-27557-1)
Supplement: Supplementary file 1 — Supplementary Information [file 41467_2021_27557_MOESM1_ESM.pdf]

Supplementary Information for

Ultra-high Thermal Stability of Sputtering Reconstructed Cu-based Catalysts

Jiafeng Yu[1], Xingtao Sun[1,2], Xin Tong[1,2], Jixin Zhang[1], Jie Li[3], Shiyan Li[1,2], Yuefeng Liu[1]*, Noritatsu Tsubaki[4]*, Takayuki Abe[5], Jian Sun[1]*

[1]Dalian National Laboratory for Clean Energy, Dalian Institute of Chemical Physics, Chinese Academy of Sciences, Dalian 116023, China

[2]University of Chinese Academy of Sciences, Beijing 100049, China

[3]School of Chemistry and Chemical Engineering, Yangzhou University, Yangzhou 225002, China

[4]Department of Applied Chemistry, School of Engineering, University of Toyama, Gofuku 3190, Toyama 930-8555, Japan

[5]Hydrogen Isotope Research Center, University of Toyama, Gofuku 3190, Toyama 930-8555, Japan

*Correspondence to: yuefeng.liu@dicp.ac.cn (Y. Liu); tsubaki@eng.u-toyama.ac.jp (N. Tsubaki); sunj@dicp.ac.cn (J. Sun)

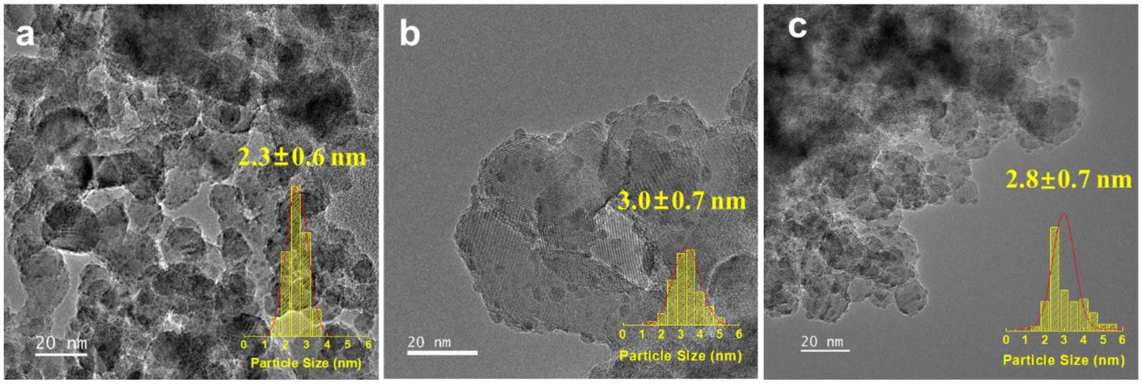

Supplementary Figure 1 | Distribution of Cu nanoparticles in different samples. TEM images and particles size distributions of (**a**) IM-Cu/TiO_2-500R, (**b**) SP-Cu/TiO_2-500R and (**c**) SP-Cu/LaTiO_2-ROR. The average particle size of Cu in different samples are 2.3, 3.0, 2.8 nm, respectively.

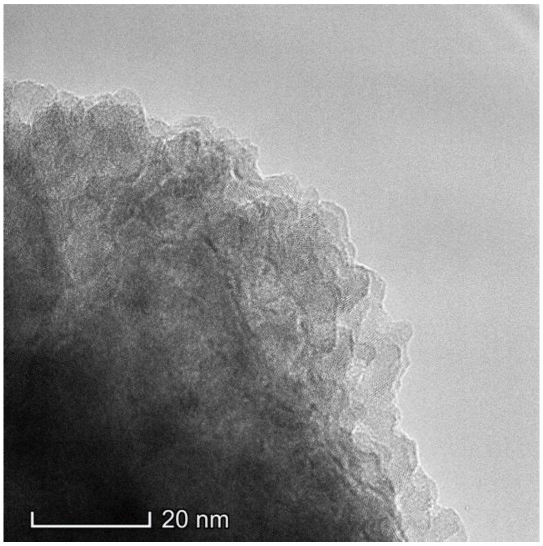

Supplementary Figure 2 | Identification of Cu nanoparticles. HRTEM images of SP-Cu/LaTiO_2-500R.

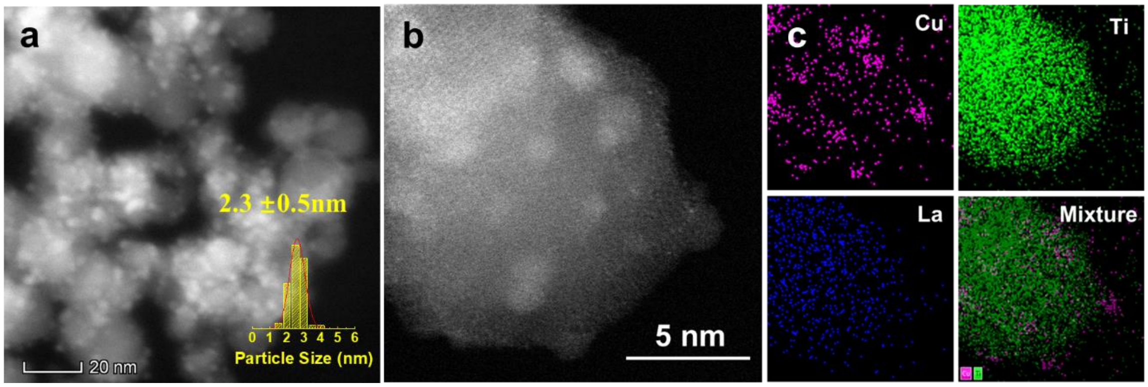

Supplementary Figure 3 | Identification of Cu nanoparticles. a, Atomic resolution annular dark-field scanning transmission electron microscope (ADF-STEM) images of SP-Cu/LaTiO_2-500R. The average Cu particle size is 2.3 nm. **b,** ADF-STEM image and **c**, element mapping analysis of SP-Cu/LaTiO_2-500R sample. Cu nanoparticles were well dispersed on LaTiO_2 support. La was also well dispersed in TiO_2 without forming large particles.

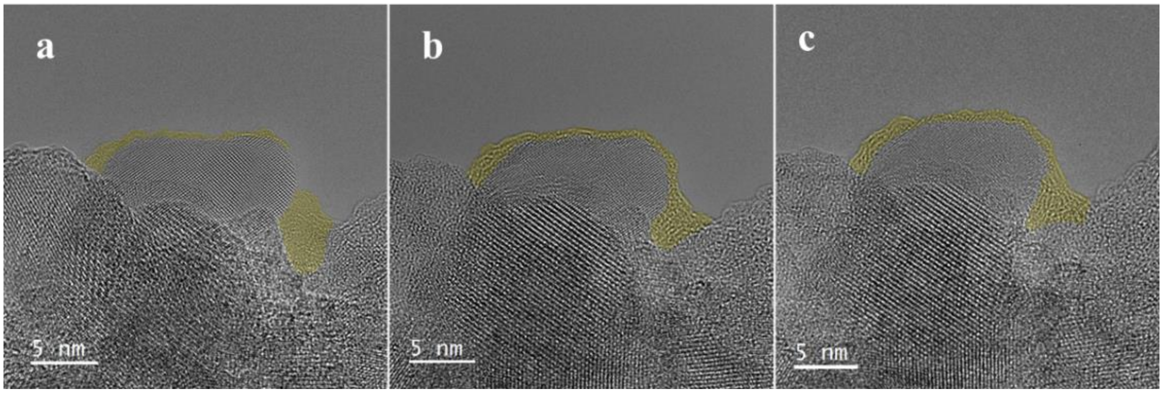

Supplementary Figure 4 | Dynamic encapsulation process. Aberration-corrected HRTEM images of dynamic encapsulation process of amorphous TiO_{2-x} (dyed yellow) on Cu particles derived by electron transmissions in SP-Cu/LaTiO_2-500R after exposure for 60 (**a**), 300 (**b**) and 600 (**c**) seconds.

Note: The induction by electron beam was dependent on the possibility of encapsulation formation, which can only promote but not create the encapsulation.

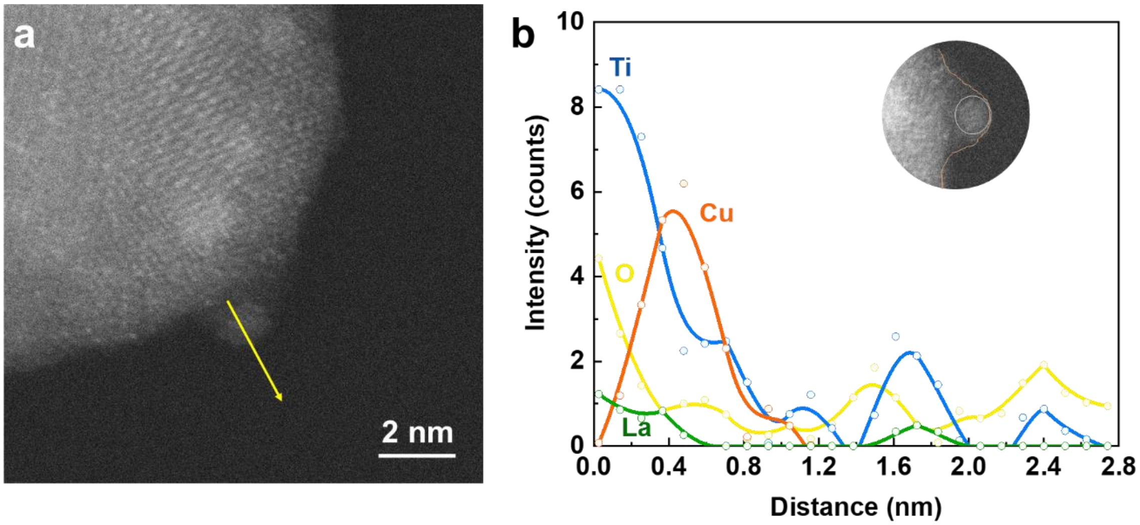

Supplementary Figure 5 | Identification of encapsulation. ADF-STEM image (**a**) and line scanning elemental analysis (**b**) of SP-Cu/LaTiO_2-500R sample. The line profile was collected along the yellow arrow in the image with a noise level of 2 counts. Ti species can be detected on half of the Cu surface, indicating that an about 0.8 nm Cu nanoparticle was encapsulated by TiO_x species as the profile described in the inset image.

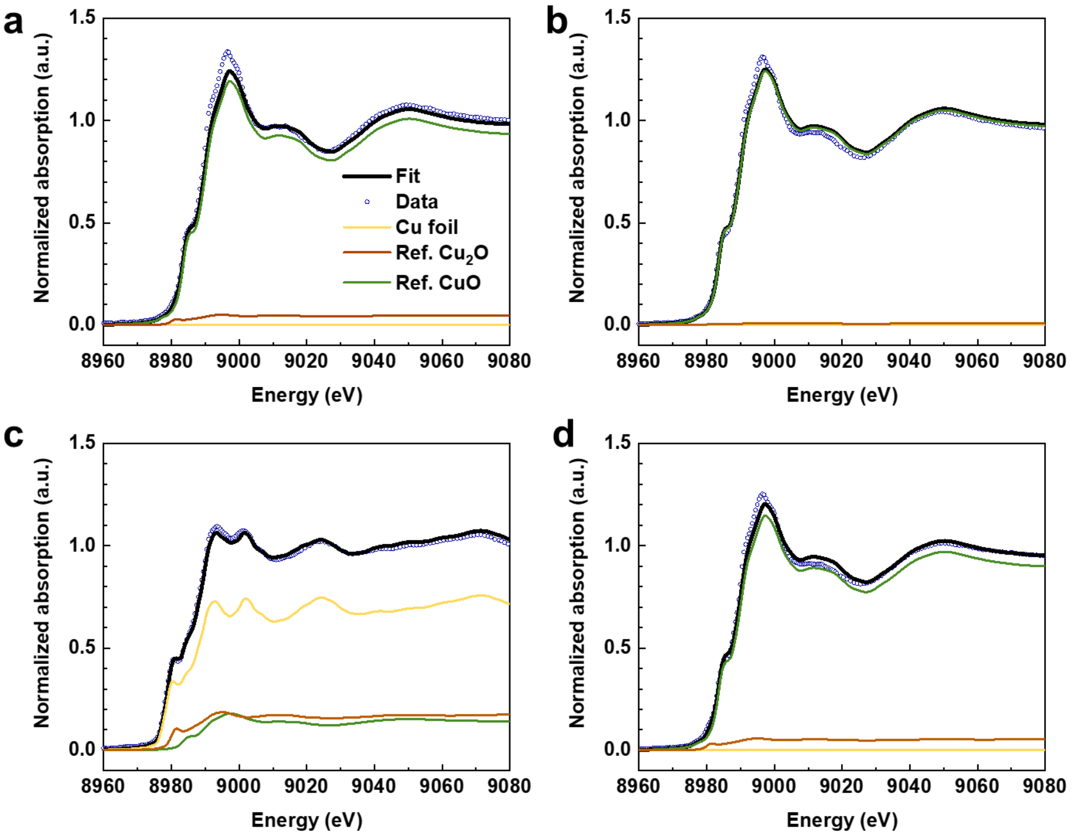

Supplementary Figure 6 | Analysis of Cu component. Linear combination fitting results of Cu K XANES data of (**a**) IM-Cu/TiO_2-500R, (**b**) SP-Cu/TiO_2-500R, (**c**) SP-Cu/LaTiO_2-500R and (**d**) SP-Cu/LaTiO_2-ROR samples after exposing to the air at room temperature for a long time. Details can be found in Supplementary Table 1.

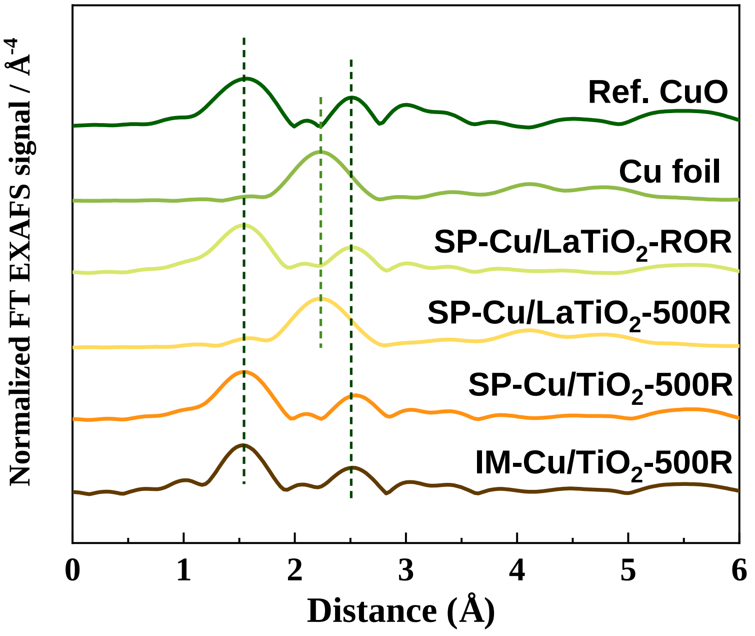

Supplementary Figure 7 | Identification of Cu species. Fourier transforms of k^3-weighted Cu K EXAFS signals without phase correction of different samples, Cu foil and reference CuO.

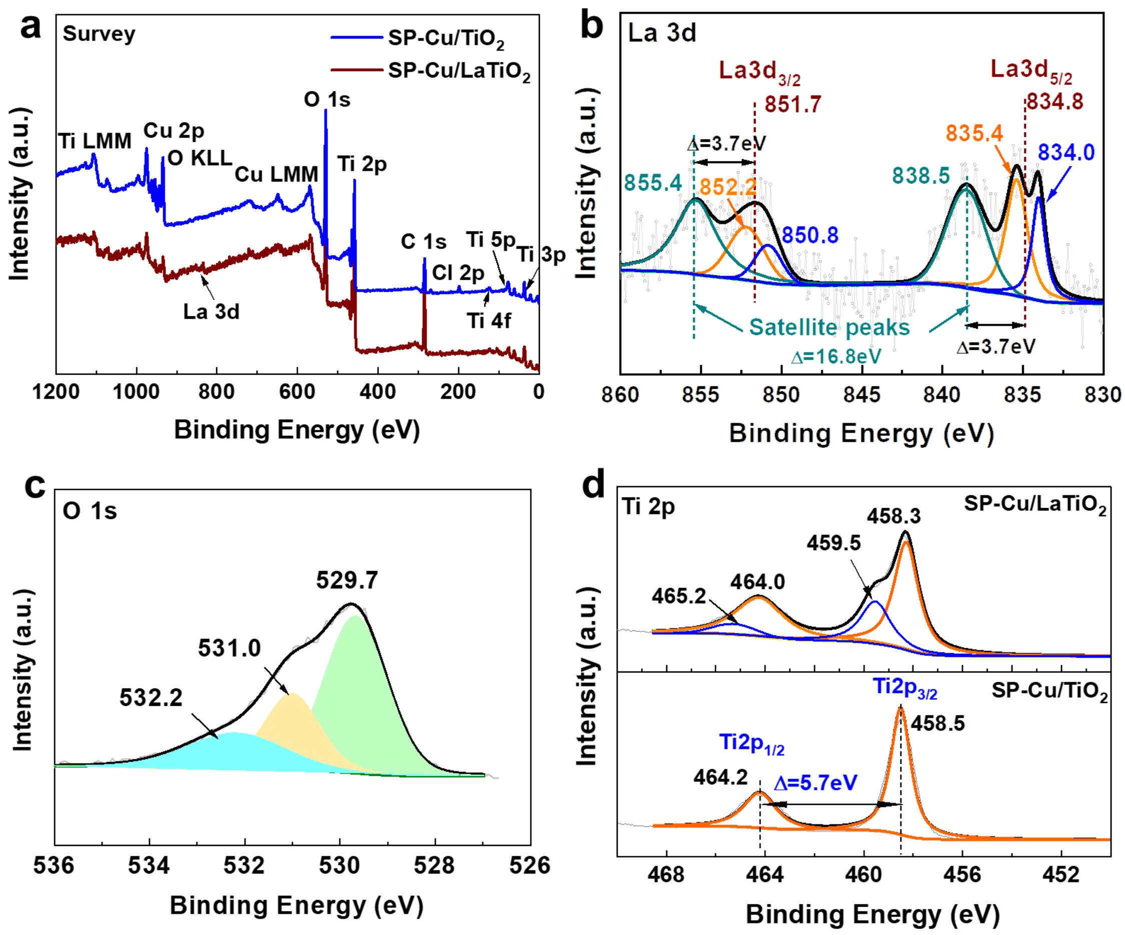

Supplementary Figure 8 | Analysis of Ti-O-La structure. Survey (**a**), La 3*d* (**b**), O 1*s* (**c**) and Ti 2*p* (**d**) XPS spectra of SP-Cu/$LaTiO_2$-fresh sample.

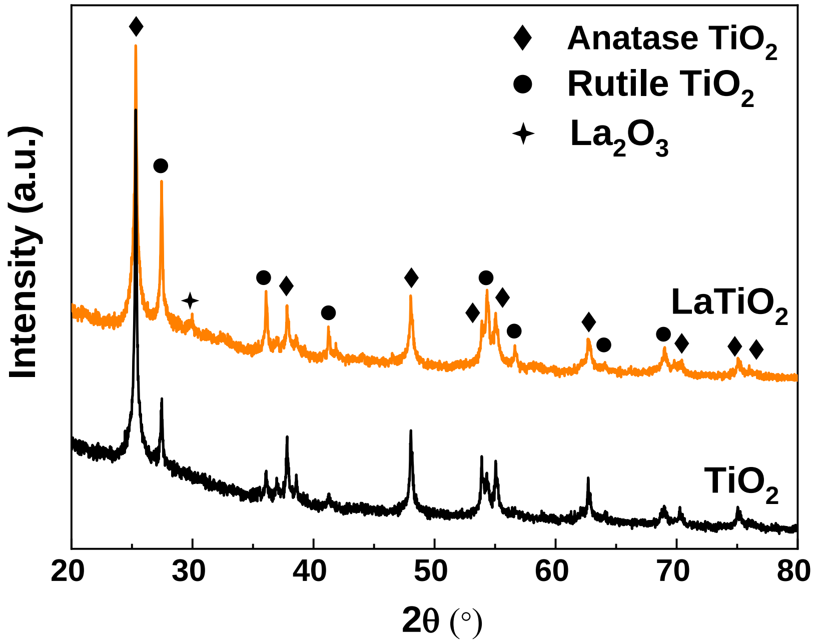

Supplementary Figure 9 | Analysis of phase component. XRD patterns of the supports: TiO_2 nanoparticles (TiO_2) and 3.8wt.% La-doped TiO_2 made by FSP ($LaTiO_2$). Usually, the amount of rutile and anatase phases were calculated by eq. $f_A=1/(1+1.26I_{R(110)}/I_{A(101)})$, where f_A is the amount of anatase in TiO_2; $I_{A(101)}$ and $I_{R(110)}$ are the peak intensity of anatase (101) and the rutile (110) in XRD, respectively. The ratio of anatase and rutile was 82% and 62 % for TiO_2 and $LaTiO_2$ support, respectively.

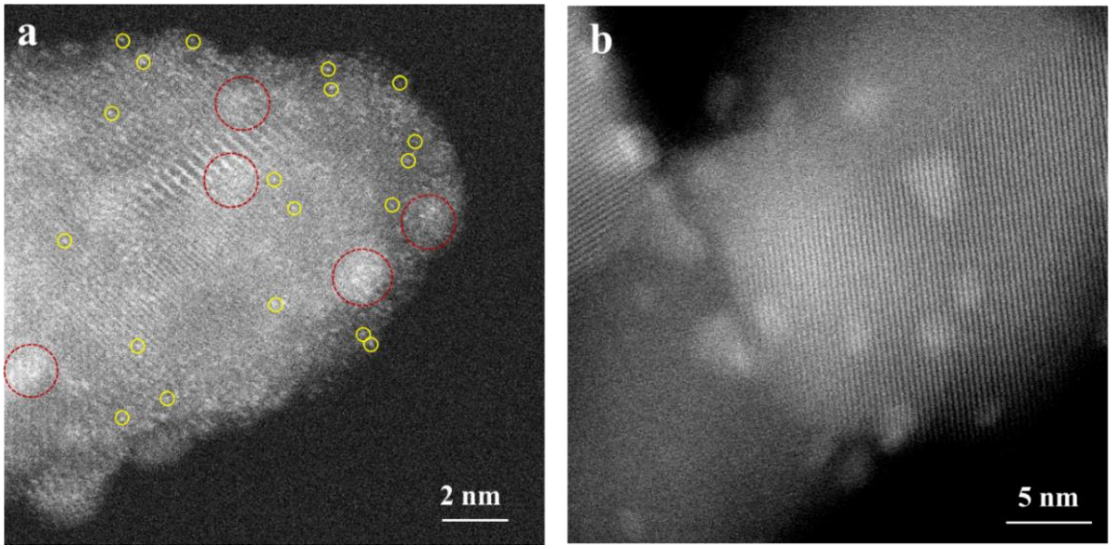

Supplementary Figure 10 | Observation of La atoms in STEM images. ADF-STEM image of (**a**) SP-Cu/LaTiO_2-500R and (**b**) SP-Cu/TiO_2-500R samples. Red marks represented Cu nanoparticles, which was confirmed by EDS elemental mapping in Supplementary Figure 2. The bright dots marked with yellow circles were assigned to the La atoms in the form of La-O-Ti structure in La-doped TiO_2 oxide compound. In comparison, no bright dots can be detected in SP-Cu/TiO_2 sample.

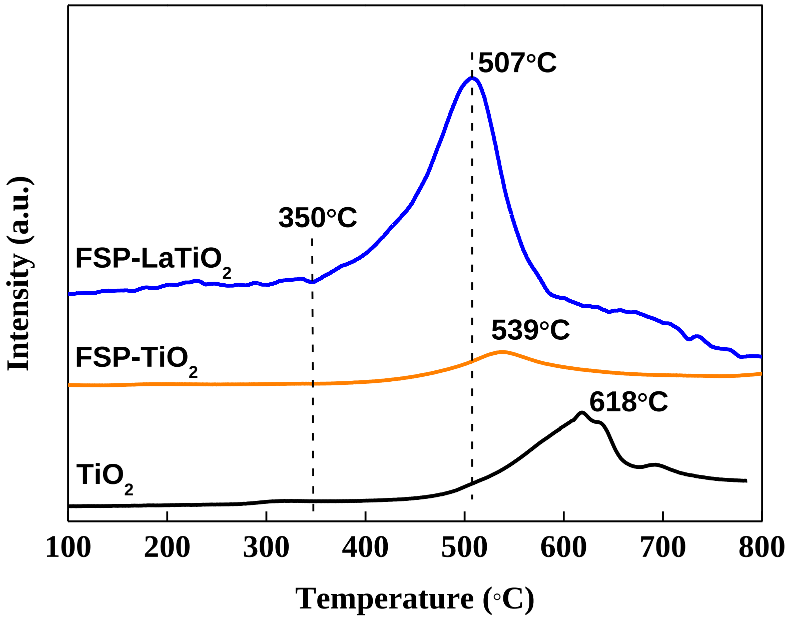

Supplementary Figure 11 | Reducibility of oxide supports. TPR results of the supports: TiO_2 nanoparticles (TiO_2), TiO_2 made by FSP (FSP-TiO_2) and 3.8 wt.% La-doped TiO_2 made by FSP (FSP-LaTiO_2).

Note: FSP method could decrease the reduction temperature of TiO_2 from 618 to 539 ℃, and it can be further declined to 507 ℃ when doping a small amount of La. The reduction process of commercial TiO_2 would not start until 500 ℃, while it already happened for FSP-LaTiO_2 at as low as 350 ℃.

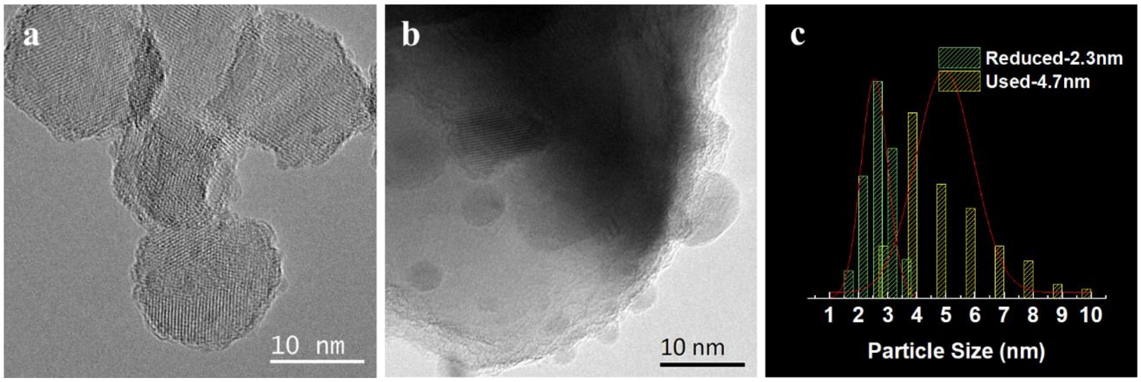

Supplementary Figure 12 | TEM image and distribution of Cu nanoparticles. HRTEM images of (**a**) IM-Cu/TiO_2 catalyst after reduction at 500°C for 1h (IM-Cu/TiO_2-500R) and (**b**) IM-Cu/TiO_2 catalyst after RWGS reaction at 600°C for 50 h (IM-Cu/TiO_2-50h), as well as (**c**) particle size distributions of reduced and used catalyst. The particle sizes of Cu in reduced and used samples were 2.3 ±0.6 and 4.7 ±1.2 nm, respectively.

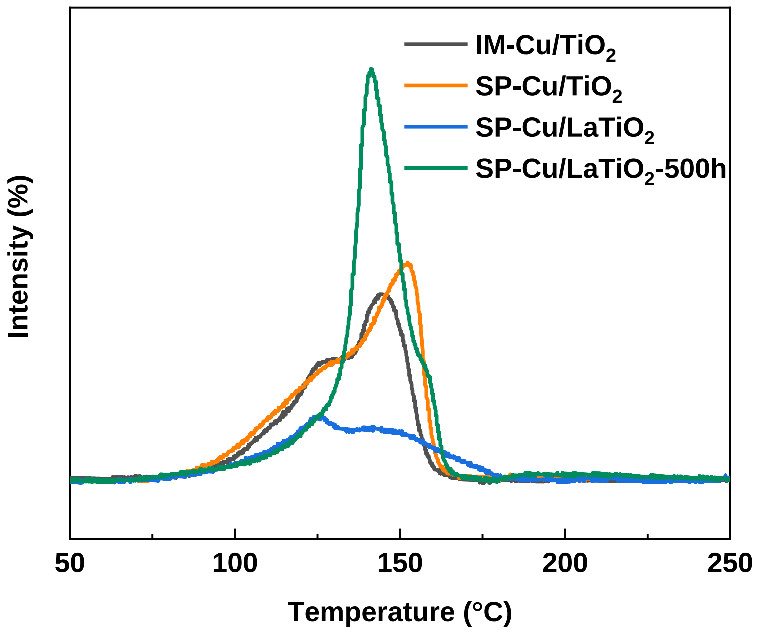

Supplementary Figure 13 | Cu dispersion measurements. TPR results after N_2O oxidation at 60 ℃ for 1 h. All samples were pretreated in H_2 at 500 ℃ for 1 h. Cu nanoparticles were re-dispersed by SMSI.

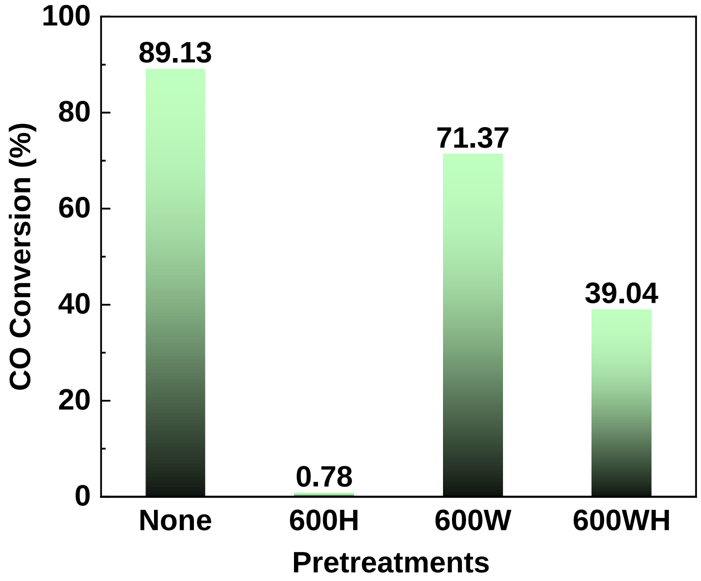

Supplementary Figure 14 | Effect of reaction atmosphere on the encapsulation. The variation of CO conversion in NO reduction by CO reaction over SP-Cu/$LaTiO_2$ after different pretreatments: 600°C in pure H_2 for 1h (600H), 600°C in 15% H_2O/He atmosphere for 1h (600W) and 600°C in 15% H_2O/H_2 atmosphere for 1h (600WH). Reaction conditions: 5%CO+5%NO+90%He, 300°C, 0.1MPa, space velocity is 15000 ml g_{cat}^{-1} h^{-1}.

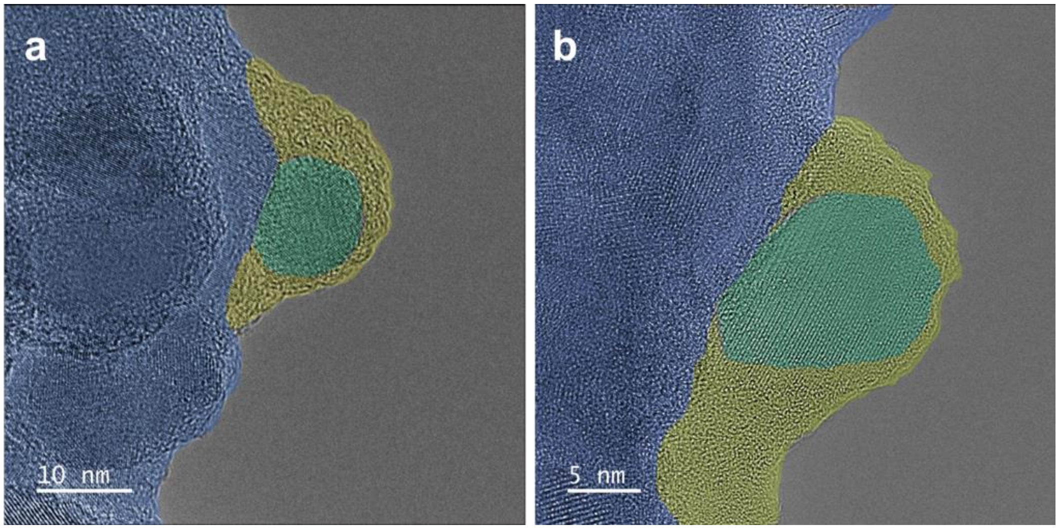

Supplementary Figure 15 | HRTEM images of large particles. a, SP-Cu/LaTiO$_2$-100h; **b,** SP-Cu/LaTiO$_2$-500h. The Cu nanoparticles, encapsulation and support were dyed with green, yellow and blue, respectively.

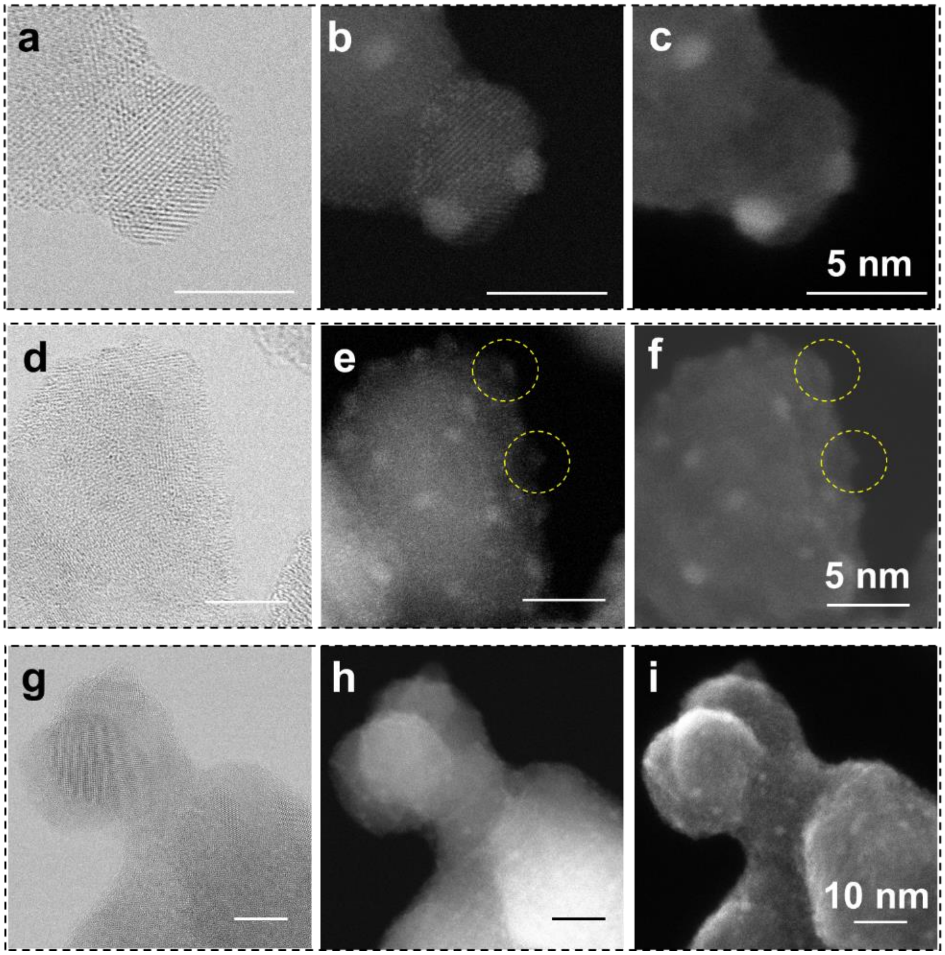

Supplementary Figure 16 | STEM images of small particles. a-c, SP-Cu/$LaTiO_2$-500R; **d-f,** SP-Cu/$LaTiO_2$-100h; **g-i,** SP-Cu/$LaTiO_2$-500h. **a,d,g** are Bright-field (BF) STEM; **b,e,h** are annular dark-field (ADF) STEM and **c,f,i** are secondary electron (SE) images. The length bar for each sample was the same as the last image. All of the images in different modes for one sample were obtained from the same part. The Cu particles on the surface of the support in ADF-STEM, marked in yellow circles, become invisible compared to its SE image, revealing that the Cu particles are partially embedded into the $LaTiO_2$ support according to the difference between ADF and SE images.

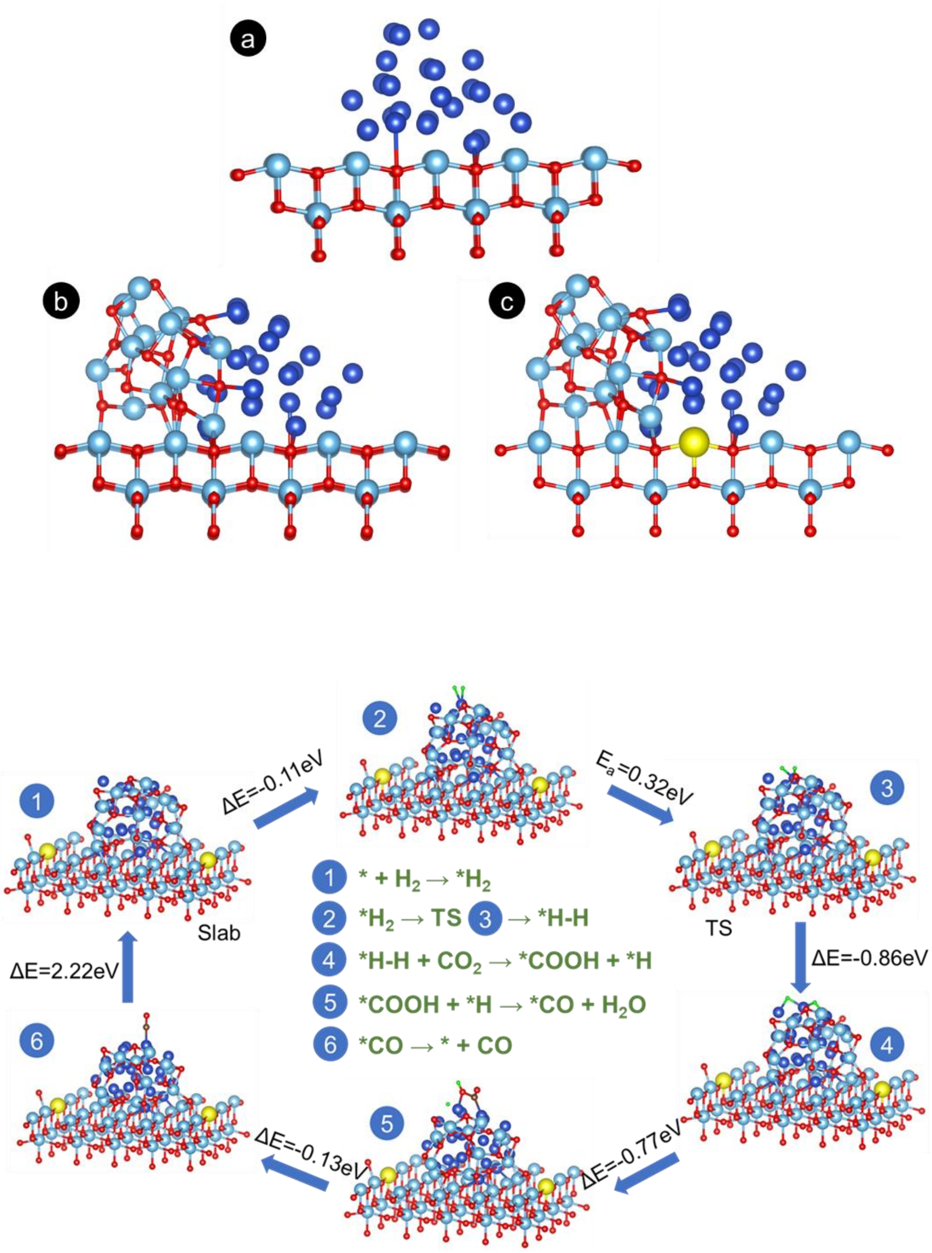

Supplementary Figure 17 | DFT calculations. Structure View (**top**) of (a) Cu_{25}/TiO_2, (b) $Cu_{25}@TiO_x/TiO_2$ and (c) $Cu_{25}@TiO_x/LaTiO_2$. The O atom (red ball), Ti atom (light blue ball), La atom (yellow ball) and Cu atom (blue ball) are shown in these structures. The RWGS reaction path (**bottom**) over $Cu_{25}@TiO_x/LaTiO_2$ model.

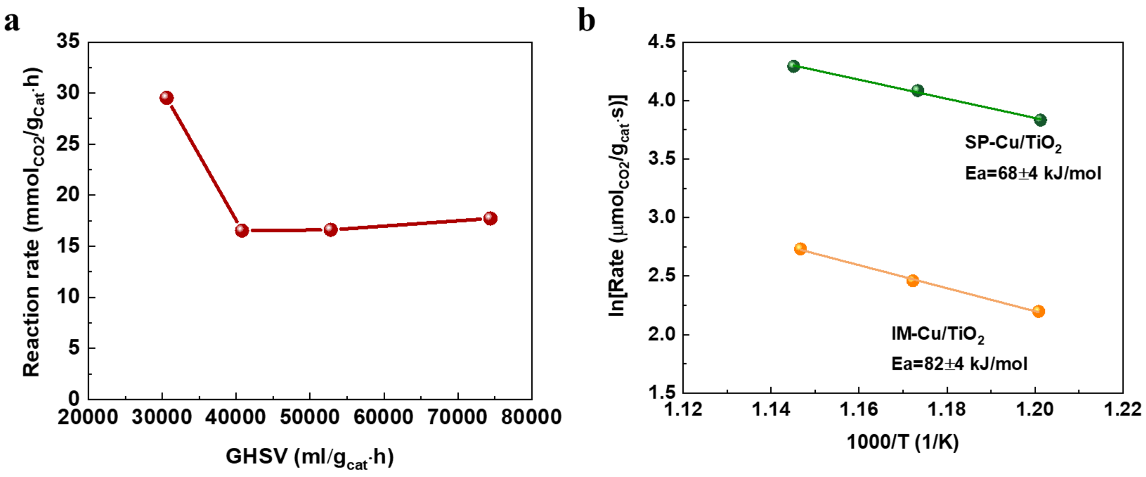

Supplementary Figure 18 | Kinetic studies. (**a**) reaction rate as a function of GHSV and (**b**) apparent activation energy (Ea) of IM-Cu/TiO_2 and SP-Cu/TiO_2 catalysts under kinetic conditions. 20 mg samples with 100 to 150 μm particle size were mixed with 80 mg quartz sand to exclude internal diffusion effects. RWGS reaction was performed at a high GHSV of 40800 ml/gcat·h to exclude external diffusion effects. The initial reaction rate was calculated according to CO conversion at 1 h. All samples were pretreated in H_2 at 500 ℃ for 1 h.

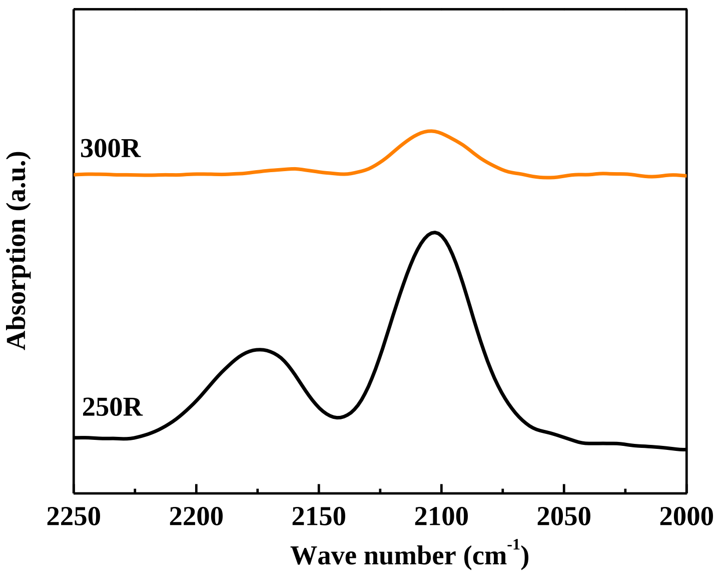

Supplementary Figure 19 | CO adsorption during reduction. IR spectra of CO adsorption over SP-Cu/$LaTiO_2$-fresh with reduction treatments in 5%H_2/He at 250 ℃ for 1h (250R) and further reduced in 5%H_2/He at 300 ℃ for 1h (300R). The spectra were obtained after CO adsorption for 30 min and the following Ar sweep for another 30 min. When increasing the reduction temperature to 300 ℃, the peak of CO adsorption on Cu oxides disappeared, meanwhile, the one for metallic Cu decreased, indicating that the formation of encapsulation has already started at this condition.

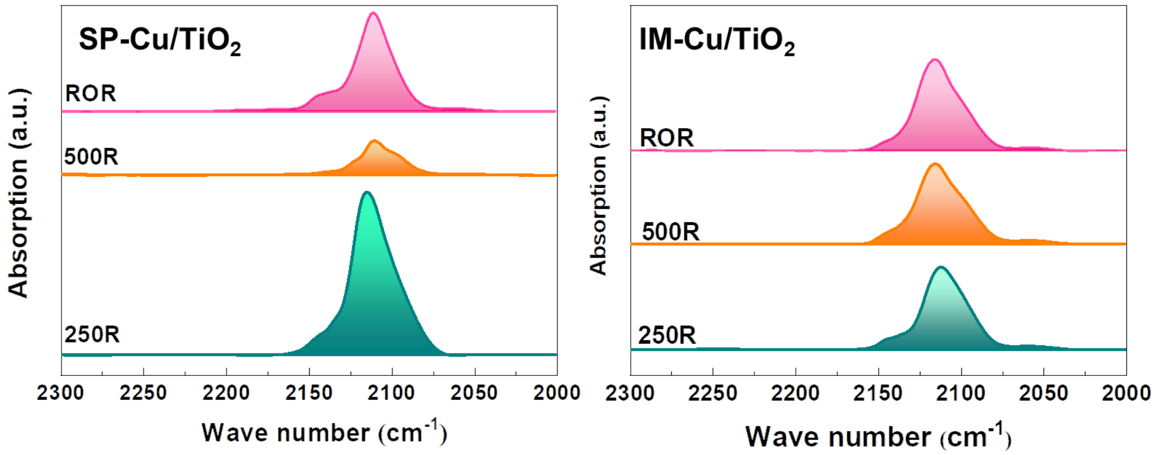

Supplementary Figure 20 | CO adsorption after different treatments. CO-IR spectra over fresh SP-Cu/TiO_2 and IM-Cu/TiO_2 samples with a successive reduction and oxidation treatments: reduced at 250 ℃ (250R), further reduced at 500 ℃ (500R), then oxidized at 400 ℃ and again reduced at 250 ℃ (ROR).

Note: After high-temperature reduction (500R), a significant decrease of CO adsorption on Cu was also found for SP-Cu/TiO_2 owing to the partial encapsulation formation. In comparison, no obvious reversible variations can be found over IM-Cu/TiO_2, excluding the existence of classical SMSI. The band close to 2150 cm^{-1} in SP-Cu/TiO_2 and IM-Cu/TiO_2 samples can be assigned to CO adsorption on the Cu species at the metal-support interface. When partial encapsulation at the interface happened on SP-Cu/TiO_2-500R as shown in Fig. 1b, the intensity was strongly weakened. After ROR treatment, the band at 2150 cm^{-1} returned. In comparison, the one at 2150 cm^{-1} was unchanged during the whole process for IM-Cu/TiO_2 sample.

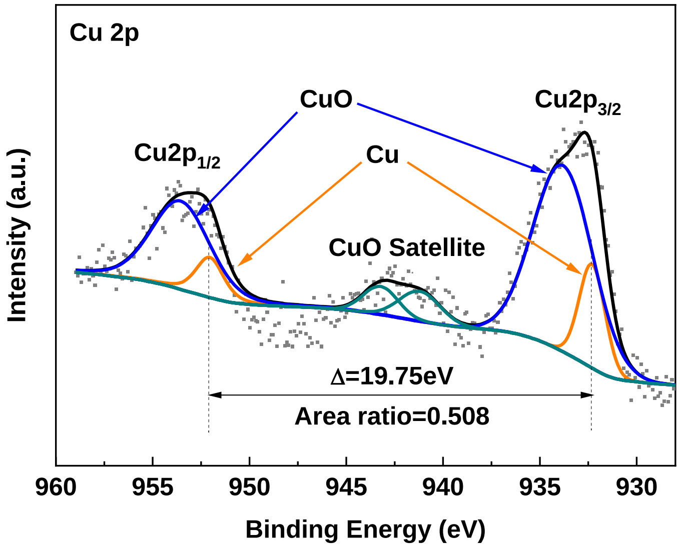

Supplementary Figure 21 | XPS measurements. The peak fitting details of Cu 2*p* XPS spectra of SP-Cu/$LaTiO_2$-fresh sample.

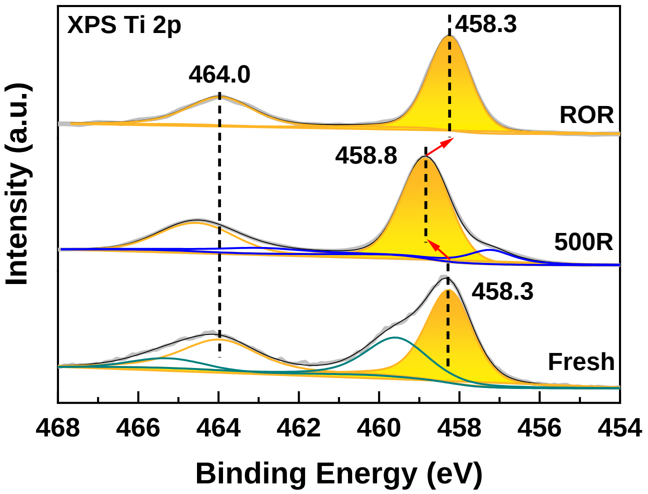

Supplementary Figure 22 | Electron transfer after different treatments. *In-situ* XPS Ti 2*p* spectra of SP-Cu/$LaTiO_2$ sample after different treatments. The fresh sample was reduced at 500 ℃ for 1 h in 99.99% H_2 flow at 0.5 MPa. The XPS spectra were measured when cooling down to room temperature (500R). Subsequently, the sample was oxidized at 400 ℃ for 1 h in 10% O_2/He flow at 0.1 MPa and cooled down to 250 ℃ for another reduction process for 1 h in 99.99% H_2 flow at 0.5 MPa. The XPS spectra were measured again after reduction (ROR).

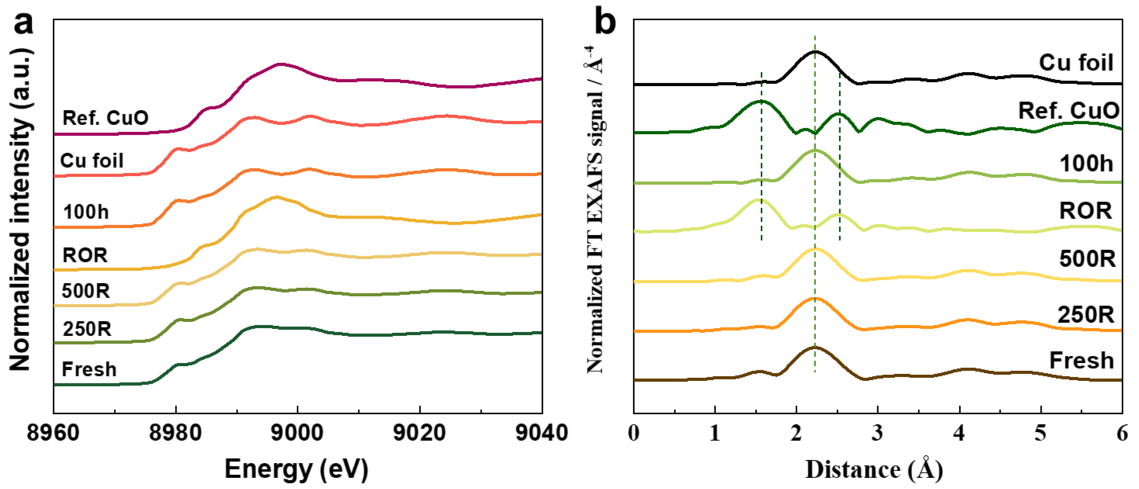

Supplementary Figure 23 | XAS analysis of Cu valence state. a, The Cu K XANES spectra of fresh SP-Cu/$LaTiO_2$, SP-Cu/$LaTiO_2$-250R, SP-Cu/$LaTiO_2$-500R, SP-Cu/$LaTiO_2$-ROR and SP-Cu/$LaTiO_2$-100h with Cu foil and CuO spectra as reference. **b,** Fourier transforms of k^3-weighted Cu K EXAFS signals without phase correction of the corresponding samples. The pre-edge of the spectrum of fresh SP-Cu/$LaTiO_2$ is similar to Cu foil rather than CuO, indicating that most of Cu existed in a metallic state in the as-prepared SP-Cu/$LaTiO_2$ sample.

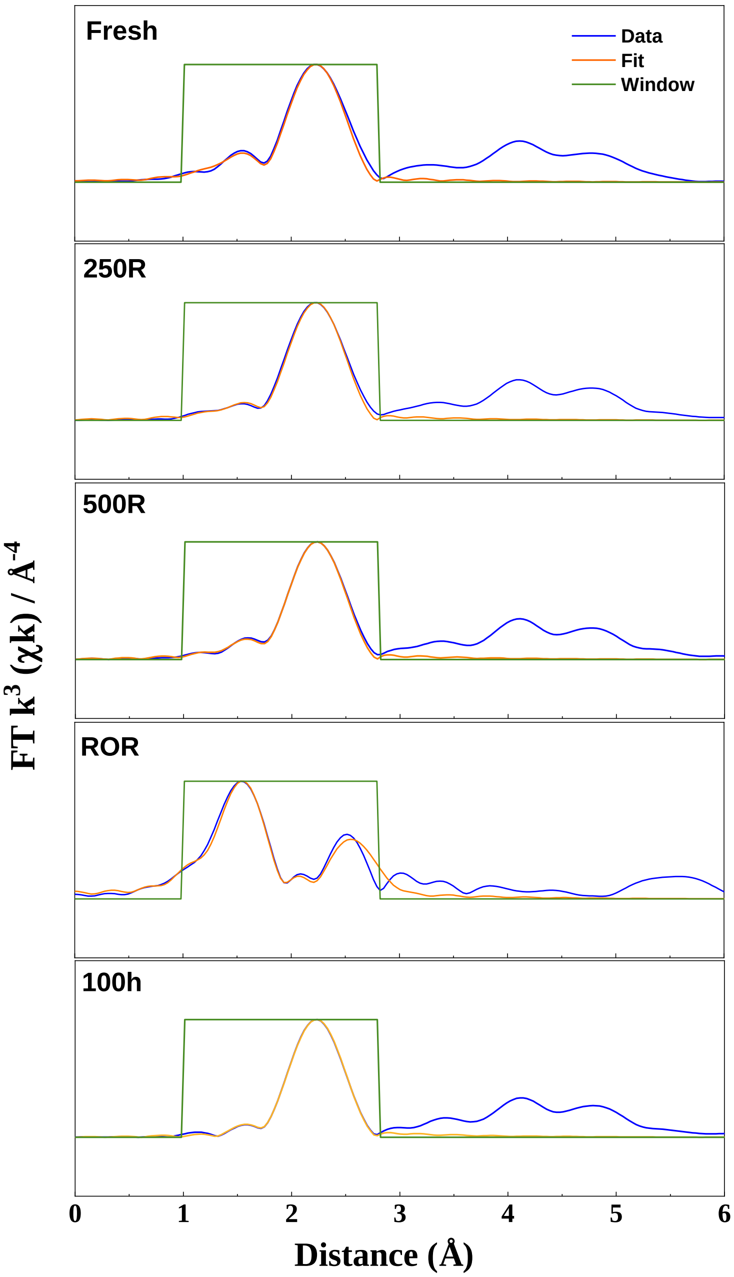

Supplementary Figure 24 | EXAFS fitting results. Cu K-edge fitting results of fresh SP-Cu/$LaTiO_2$, SP-Cu/$LaTiO_2$-250R, SP-Cu/$LaTiO_2$-500R, SP-Cu/$LaTiO_2$-ROR and SP-Cu/$LaTiO_2$-100h. Details of EXAFS fitting can be found in Supplementary Table 5.

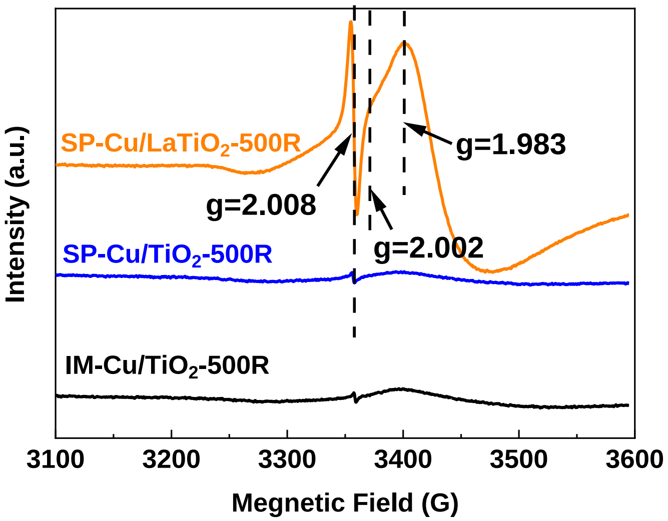

Supplementary Figure 25 | EPR results. EPR spectra of tested samples after reduction at 500 ℃. EPR spectra were measured at 100 K.

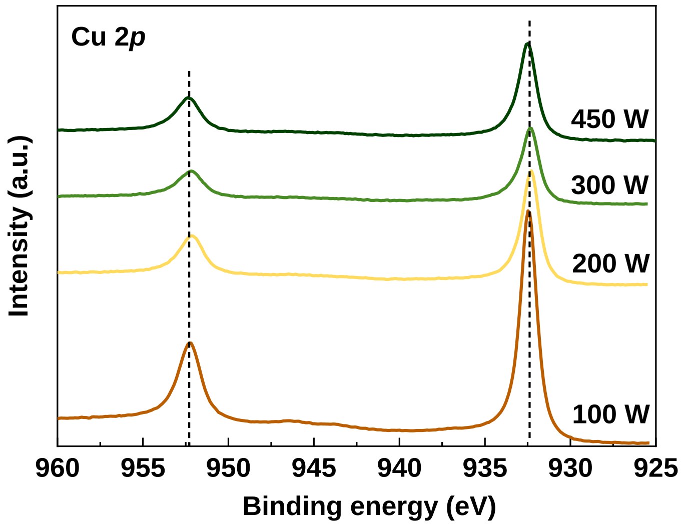

Supplementary Figure 26 | XPS analysis. Cu 2*p* XPS spectra on SP-Cu/$LaTiO_2$ catalyst prepared with different sputtering power.

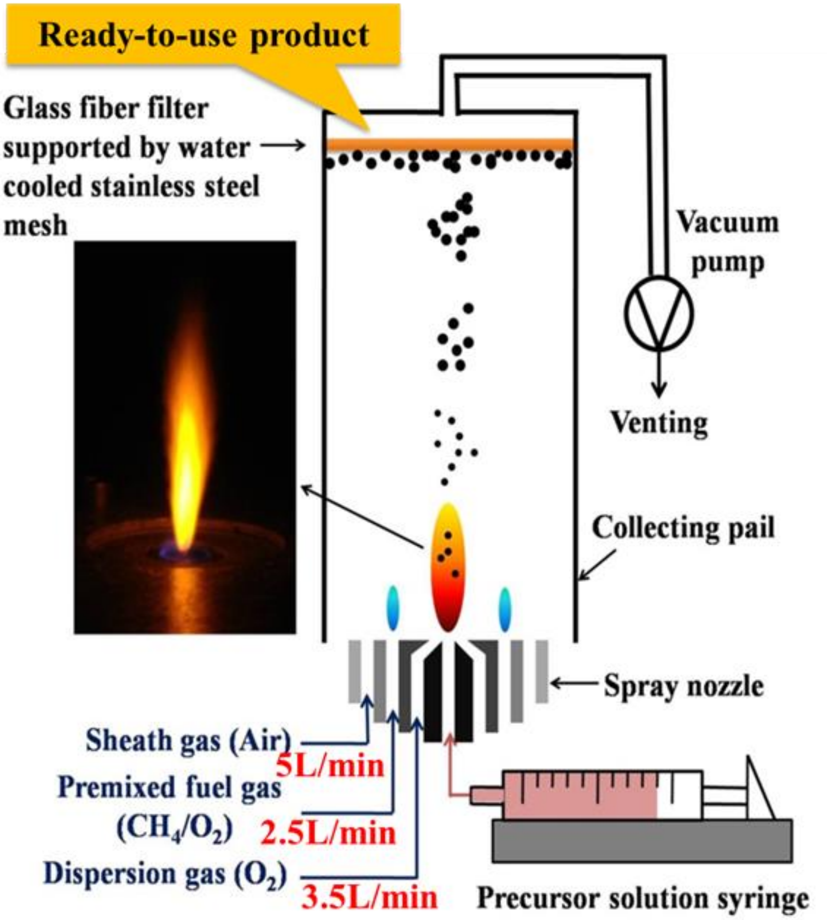

Supplementary Figure 27 | Schematic diagram of FSP setup. A typical FSP setup consisted of precursor injet, gas flow, spray nozzle and collection systems with a real image of a burning flame.

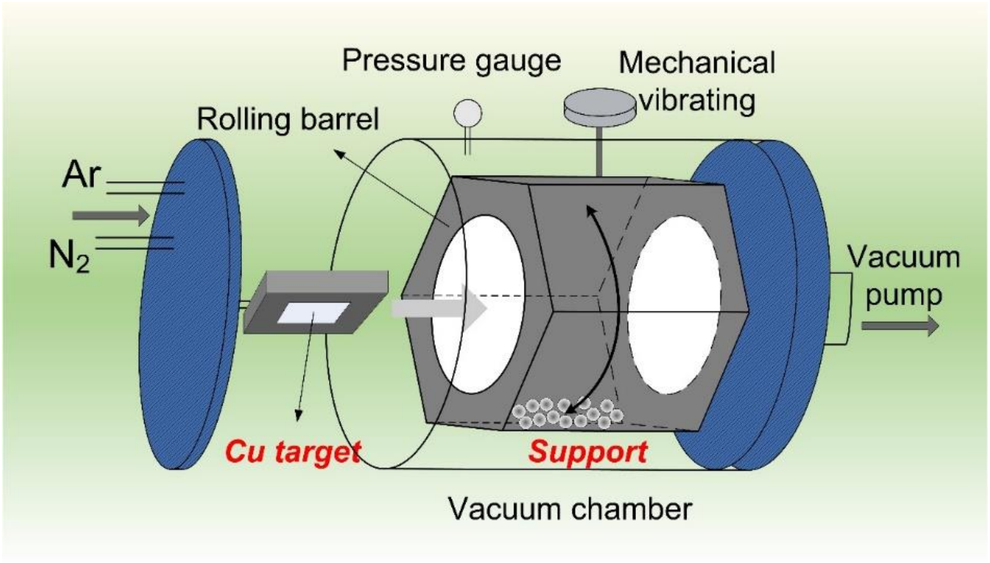

Supplementary Figure 28 | Schematic diagram of SP setup. A typical SP setup consisted of sputtering source, vacuum chamber, metal target and rotating cylinder systems.

Supplementary Table 1 | Analysis of Cu component. Linear combination fitting results of Cu K XANES data of IM-Cu/TiO_2-500R, SP-Cu/TiO_2-500R, SP-Cu/LaTiO_2-500R and SP-Cu/LaTiO_2-ROR samples after exposing to the air at room temperature for a long time. The formation of an encapsulation layer in SP-Cu/LaTiO_2-500R could protect metallic Cu from being oxidized. The uncertainty of the fitting is ±5%.

Samples	Cu (%)	Cu_2O (%)	CuO (%)	R factor
IM-Cu/TiO_2-500R	0.0	4.7	95.3	0.006
SP-Cu/TiO_2-500R	0.0	0.8	99.2	0.006
SP-Cu/LaTiO_2-500R	70.1	17.2	14.4	0.002
SP-Cu/LaTiO_2-ROR	0.0	5.3	94.7	0.005

Supplementary Table 2 | Structure parameters extracted from the EXAFS fitting. Fitting results of Cu K EXAFS data of IM-Cu/TiO_2-500R, SP-Cu/TiO_2-500R, SP-Cu/LaTiO_2-500R and SP-Cu/LaTiO_2-ROR samples. Reference materials: Cu-O in CuO at 1.90 Å (CN=6), Cu-Cu in Cu at 2.54 Å (CN=12) and Cu-O-Cu in CuO at 2.96 Å (CN=8). *k* range: 3-12Å, spline range: 0-12Å, *k*-weight=3, distance range: 1-2.8Å. S_0^2 was fixed at 0.943, and E_0 was defined as a global fit parameter for each sample. All samples were tested in the air.

Sample	Shell	CN	Distance /Å	$\sigma^2 \times 10^{-3}$/Å^2	δE_0 / eV	R factor
IM-Cu/TiO_2-500R	Cu-O	5.3±0.5	1.95±0.01	5.4±1.5	6.6±1.3	0.012
	Cu-Cu*	-	-	-	-	-
	Cu-O-Cu	6.0±2.3	2.93±0.03	10.7±1.5	6.6±1.3	0.012
SP-Cu/TiO_2-500R	Cu-O	5.9±0.5	1.95±0.00	5.9±1.3	6.8±1.0	0.008
	Cu-Cu*	-	-	-	-	-
	Cu-O-Cu	7.2±2.2	2.95±0.05	11.8±1.3	6.8±1.0	0.008
SP-Cu/LaTiO_2-500R	Cu-O	1.0±0.2	1.90±0.05	5.2±0.4	2.4±0.8	0.002
	Cu-Cu	9.7±0.7	2.54±0.01	10.4±0.4	2.4±0.8	0.002
	Cu-O-Cu	2.2±1.5	2.89±0.01	10.4±0.4	2.4±0.8	0.002
SP-Cu/LaTiO_2-ROR	Cu-O	5.5±0.4	1.95±0.01	6.4±1.3	6.4±1.1	0.007
	Cu-Cu	0.9±0.6	2.58±0.03	12.9±1.3	6.4±1.1	0.007
	Cu-O-Cu	6.6±2.1	2.93±0.03	12.9±1.3	6.4±1.1	0.007

*****No reliable fitted values can be obtained for this CN

Supplementary Table 3 | Comparison of Cu property and activity on various TiO_2 support. Property of Cu in IM-Cu/TiO_2, SP-Cu/TiO_2 and SP-Cu/LaTiO_2 after being pretreated in H_2 at 500 ℃ for 1 h (-500R).

Catalysts	Cu loading[a] (wt.%)	Exposed Cu surface area[b] (m^2/g)	Cu dispersion (%)	Reaction rate[c] ($mmol_{CO2}/m^2_{Cu}\cdot h$)	TOF[d] (s^{-1})
IM-Cu/TiO_2-500R	12.6	28.8	35.4	1.9	0.02
SP-Cu/TiO_2-500R	18.7	28.3	23.5	9.3	0.11
SP-Cu/LaTiO_2-500R	14.6	12.7	13.5	1.9	0.05

[a]Cu loading was identified by XPS.

[b]The exposed Cu surface area was measured by TPR after N_2O oxidation at 60 ℃ for 1 h.

[c]The reaction rate was calculated according to the CO_2 conversion at 2 h. The metallic Cu surface area used here was computed based on an atomic copper surface density of 1.46×10^{19} Cu atoms per m^2.

[d]TOF was calculated based on the reaction rate measured under kinetics conditions at 1h.

Supplementary Table 4 | DFT calculation. Comparison of reaction path and energy difference in RWGS reaction over Cu_{25}/TiO_2, $Cu_{25}@TiO_x/TiO_2$ and $Cu_{25}@TiO_x/LaTiO_2$.

Order	Reaction path	Energy difference (ΔE /eV)		
		Cu_{25}/TiO_2	$Cu_{25}@TiO_x/TiO_2$	$Cu_{25}@TiO_x/LaTiO_2$
1	$*+H_2 \rightarrow *H_2$	-0.27	-0.15	-0.11
2	$*H_2 \rightarrow TS$	0.63	0.28	0.32
3	$TS* \rightarrow H\text{-}H$	-0.67	-0.86	-0.80
4	$*H\text{-}H+CO_2 \rightarrow *COOH+*H$	-0.09	-0.96	-0.77
5	$*COOH+*H \rightarrow *CO+H_2O$	0.02	-0.16	-0.13
6	$*CO \rightarrow *+CO$	1.11	2.58	2.22

Supplementary Table 5 | Structure parameters extracted from the EXAFS fitting. Fitting results of Cu K EXAFS data of SP-Cu/$LaTiO_2$-fresh, SP-Cu/$LaTiO_2$-250R, SP-Cu/$LaTiO_2$-500R, SP-Cu/$LaTiO_2$-ROR and SP-Cu/$LaTiO_2$-100h samples. Reference materials: Cu-O in CuO at 1.90 Å (CN=6), Cu-Cu in Cu at 2.54 Å (CN=12) and Cu-O-Cu in CuO at 2.88 Å (CN=8). Each sample was fitted in the same model with the following parameters. *k* range: 3-12Å, spline range: 0-12Å, *k*-weight=3, distance range: 1-2.8Å. S_0^2 was fixed at 0.943, and E_0 was defined as a global fit parameter for each sample. All of the samples were exposed to the air during EXAFS measurements.

Sample	Path	CN	Distance/Å	σ^2×10^{-3}/Å^2	ΔE_0/eV	R factor
SP-Cu/$LaTiO_2$-as prepared	Cu-O	1.6±0.2	1.88±0.08	5.8±0.5	2.0±1.2	0.002
	Cu-Cu	8.3±0.9	2.54±0.02	11.6±0.5	2.0±1.2	0.002
	Cu-O-Cu	3.9±1.9	2.88±0.02	11.6±0.5	2.0±1.2	0.002
SP-Cu/$LaTiO_2$-250R	Cu-O	0.8±0.2	1.86±0.09	5.2±0.3	2.1±1.0	0.003
	Cu-Cu	8.1±0.8	2.54±0.02	10.4±0.3	2.1±1.0	0.003
	Cu-O-Cu	2.0±1.6	2.88±0.02	10.4±0.3	2.1±1.0	0.003
SP-Cu/$LaTiO_2$-500R	Cu-O	1.0±0.2	1.90±0.05	5.2±0.4	2.4±0.8	0.002
	Cu-Cu	9.7±0.7	2.54±0.01	10.4±0.4	2.4±0.8	0.002
	Cu-O-Cu	2.2±1.5	2.89±0.01	10.4±0.4	2.4±0.8	0.002
	Cu-O	5.5±0.4	1.95±0.01	6.4±1.3	6.4±1.1	0.007
SP-CuLaTi-ROR	Cu-Cu	0.9±0.6	2.58±0.03	12.9±1.3	6.4±1.1	0.007
	Cu-O-Cu	6.6±2.1	2.93±0.03	12.9±1.3	6.4±1.1	0.007
SP-Cu/$LaTiO_2$-100h	Cu-O*	-	-	-	-	-
	Cu-Cu	9.7±0.6	2.54±0.02	8.7±1.3	3.5±0.7	0.001
	Cu-O-Cu*	-	-	-	-	-

*No reliable fitted values can be obtained for these paths.

Supplementary Table 6 | Peak information of Auger electron spectra. AES results of SP-Cu/$LaTiO_2$ catalysts prepared with different sputtering power.

Sputtering Power (W)	Peak-1 (eV)	Peak-2 (eV)	Peak-3 (eV)	Peak-4 (eV)
100	921.3	918.6	916.6	913.7
200	921.5	918.8	916.7	913.7
300	921.7	918.9	916.8	913.7
450	921.7	918.9	916.8	913.7
